# 

**Published:** 1899-07-08

**Authors:** 


**Notices of Births, Deaths, and Marriages** are inserted in this column at a charge of 2s. 6d. for an announcement not exceeding four lines.

### NOTICE TO CORRESPONDENTS.

All MS., Letters, Books for Review, and other matters intended for the Editor should be addressed The Editor, "The Hospital," The Hospital Building, 28 & 29, Southampton Street, Strand, W.C.

The Editor cannot undertake to return rejected MS., even when accompanied by stamped directed envelope.

**Notice to Advertisers and Subscribers.**

All Advertisements, Orders for Copies of The Hospital, and Business Communications should be addressed to **THE MANAGER (Not to the Editor),** The Hospital, The Hospital Building, 28 & 29, Southampton Street, Strand, London, W.C.

The Cost of Subscription, post free, is as follows:—Per week, 2½d.; per quarter, 2s. 8d.; per half-year, 5s. 3d.; per annum, 10s. 6d. Foreign:—Per annum, 15s. 2d., payable, in all cases, in advance.

**NOTICE.—Vol. XXV. of "The Hospital" (October, 1898, to March, 1899), in handsome cloth binding, is now on sale at the Office of this Journal, price 7s. 6d. Cases for binding the Numbers contained in this Volume 1s. 6d. Index to Vol. XXV., 2½d., post free.**

**The Monthly Part for July is now ready. Price 6d., by post 8d.**

### BOOKS RECEIVED.

The F. A. Davis Co.

"Diseases of the Ear, Nose, and Throat." By Seth Scott Bishop, M.D., D.C.L., LL.D.

Alden and Co.

"The Radcliffe Infirmary, Oxford." By E. Vaughan Jenkins.

T. Fisher Unwin.

"Claude Bernard." Masters of Medicine Series. By Michael Foster M.A., M.D., D.C.L.

Sampson Low, Marston, and Co.

"Twentieth Century Practice of Modern Medical Science." Edited by Thomas L. Stedman, M.D. Volume XVI.

John Wright and Co.

"An Introduction to Dermatology." By Norman Walker, M.D.
"Our Baby." By Mrs. Langton Hewer.

Scientific Press.

"Chats about the Microscope." By Henry C. Shelley.
The Pocket Case Book for District and Private Nurses.

H. K. Lewis.

"Massage and the Original Swedish Movements." By Kurre W. Ostrom.

The Medical Publishing Co.

"Pyorrhœa Alveolaris and its Relation to General Medicine." By John Fitzgerald, L.D.S.

Amateur Gardening Offices.

"Amateur Gardening." Vol. XV.

The People's Journal.

"Aunt Katie's Mothers' Guide."

F. Nash and Co., Bournemouth.

"Reports of the Medical Officer of Health, 1898."

W. Mate and Sons, Bournemouth.

"Bournemouth Health Statistics."

Samuel Sidders and Co.

"Annual Report." By T. Orme Dudfield, M.D., Medical Officer of Health for St. Mary Abbotts, Kensington.

### THE STUDENT OF MEDICINE.

### APPOINTMENTS.

A. Berrill, L.R.C.P.Lond., M.R.C.S.Eng., appointed Medical Officer for the Woodford District of the West Ham Union. F. P. Cayley, M.R.C.S., L.R.C.P., appointed Assistant Medical Officer at the King's Road Workhouse of the Parish of St. Pancras. E. C. Drake, L.S.A., appointed Medical Officer for the Tudhoe District of the Durham Union. W. Faull, L.R.C.P., L.R.C.S.Edin., appointed House Surgeon to the Dewsbury Infirmary. W. J. Foster, F.R.C.S., Q.K.C.P., appointed Assistant Surgeon to the Royal Berks Hospital, Reading. A. H. Griffith, M.D.Aberd., F.R.C.S.Edin., appointed Honorary Ophthalmic Surgeon to the Manchester Royal Infirmary. J. J. Hanly, L.R.C.P., L.R.C.S.Edin., appointed Medical Officer for the Second District of the Shepton Mallet Union. B. Hogan, L.S.A., appointed Medical Officer for the Bromley Workhouse of the Stepney Union. A. G. Lawrence, M.D.St.And., M.R.C.S., appointed Medical Officer for the Shirenewton District of the Chepstow Union. C. H. Leaf, M.A., M.B.Cantab., F.R.C.S.Eng., appointed Surgeon to the Out-patients at the Gordon Hospital for Diseases of the Rectum. C. G. MacVicker, B.A., M.B., appointed Medical Officer and Public Vaccinator for the Fourth District Wells Union. F. S. Pitt-Taylor, M.B., Ch.B.Vict., appointed Assistant Medical Officer to Mill Road Infirmary, Liverpool. A. G. Reid, B.Sc.Lond., M.B., C.M.Edin., appointed Honorary Surgeon to the Rotherham Hospital. E. S. Reynolds, M.D., F.R.C.P.Lond., appointed Honorary Assistant Physician to the Manchester Royal Infirmary. G. A. Turner, M.B., D.P.H., appointed Assistant Medical Officer to Grahamstown Asylum, South Africa. C. E. Wallis, M.R.C.S., L.R.C.P., L.D.S., appointed Assistant Dental Surgeon at King's College Hospital.

